# Supplementary material for: New insights into genome annotation in Podospora anserina through re-exploiting multiple RNA-seq data
Source: BMC Genomics. 2022 Dec 29;23:859. doi: 10.1186/s12864-022-09085-4 (PMC9801653; doi:10.1186/s12864-022-09085-4)
Supplement: Supplementary file 2 — Additional file 2. (PPTX 85 kb) [file 12864_2022_9085_MOESM2_ESM.pptx]

## Slide 1
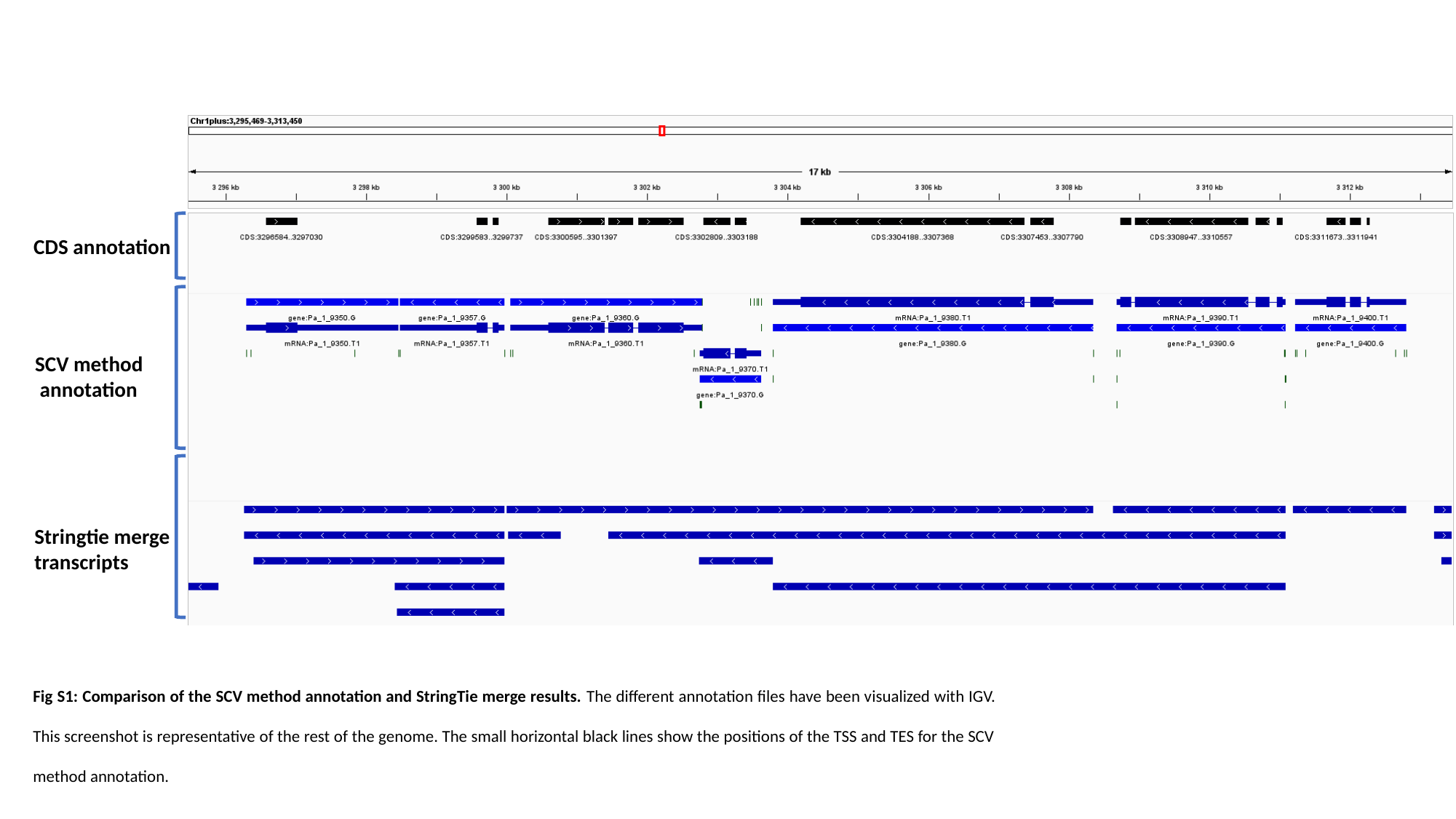

CDS annotation
SCV method
 annotation
Stringtie merge
transcripts
Fig S1: Comparison of the SCV method annotation and StringTie merge results. The different annotation files have been visualized with IGV. This screenshot is representative of the rest of the genome. The small horizontal black lines show the positions of the TSS and TES for the SCV method annotation.
