# Supplementary material for: New insights into genome annotation in Podospora anserina through re-exploiting multiple RNA-seq data
Source: BMC Genomics. 2022 Dec 29;23:859. doi: 10.1186/s12864-022-09085-4 (PMC9801653; doi:10.1186/s12864-022-09085-4)

## Slide 1
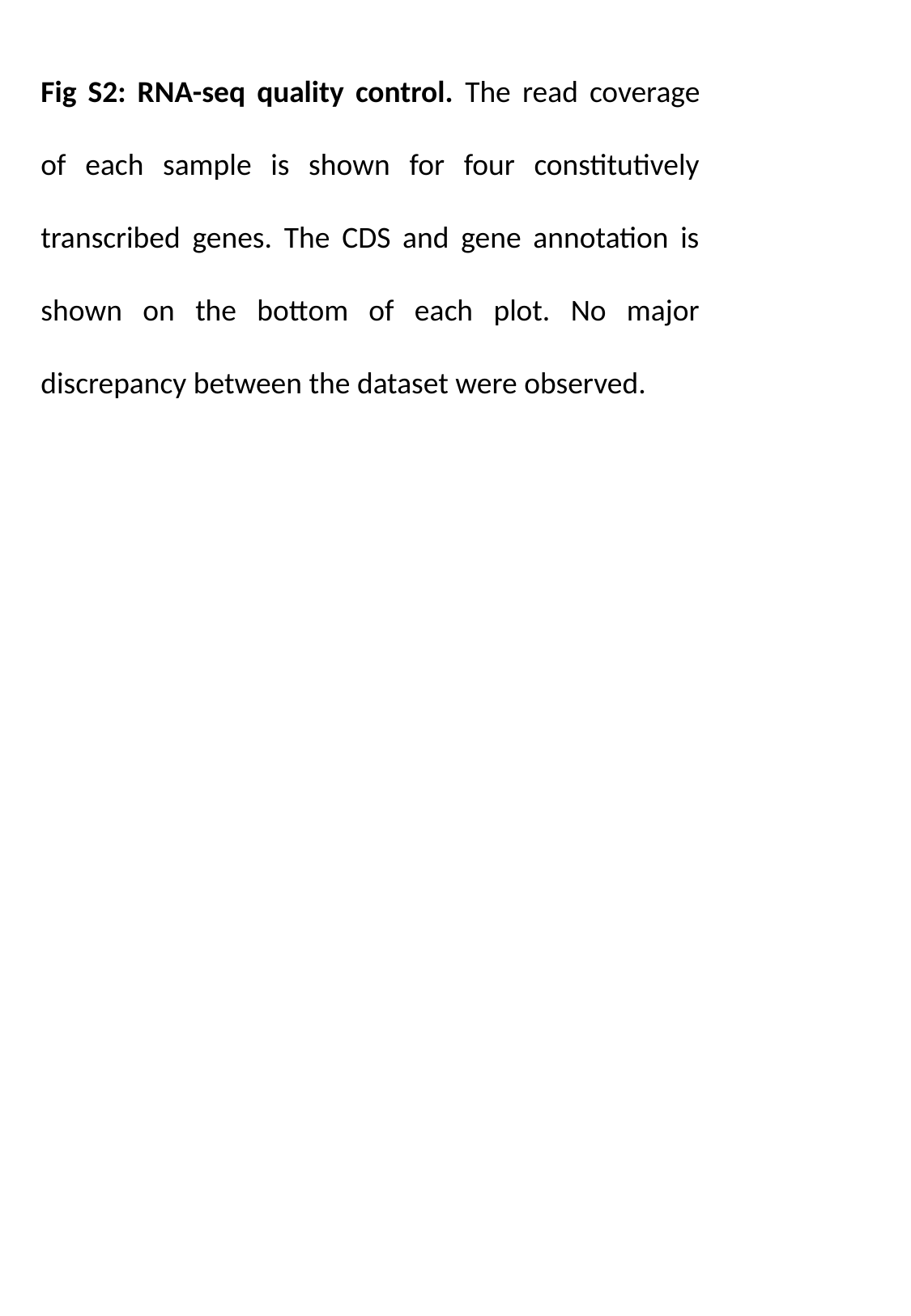

Fig S2: RNA-seq quality control. The read coverage of each sample is shown for four constitutively transcribed genes. The CDS and gene annotation is shown on the bottom of each plot. No major discrepancy between the dataset were observed.

## Slide 2
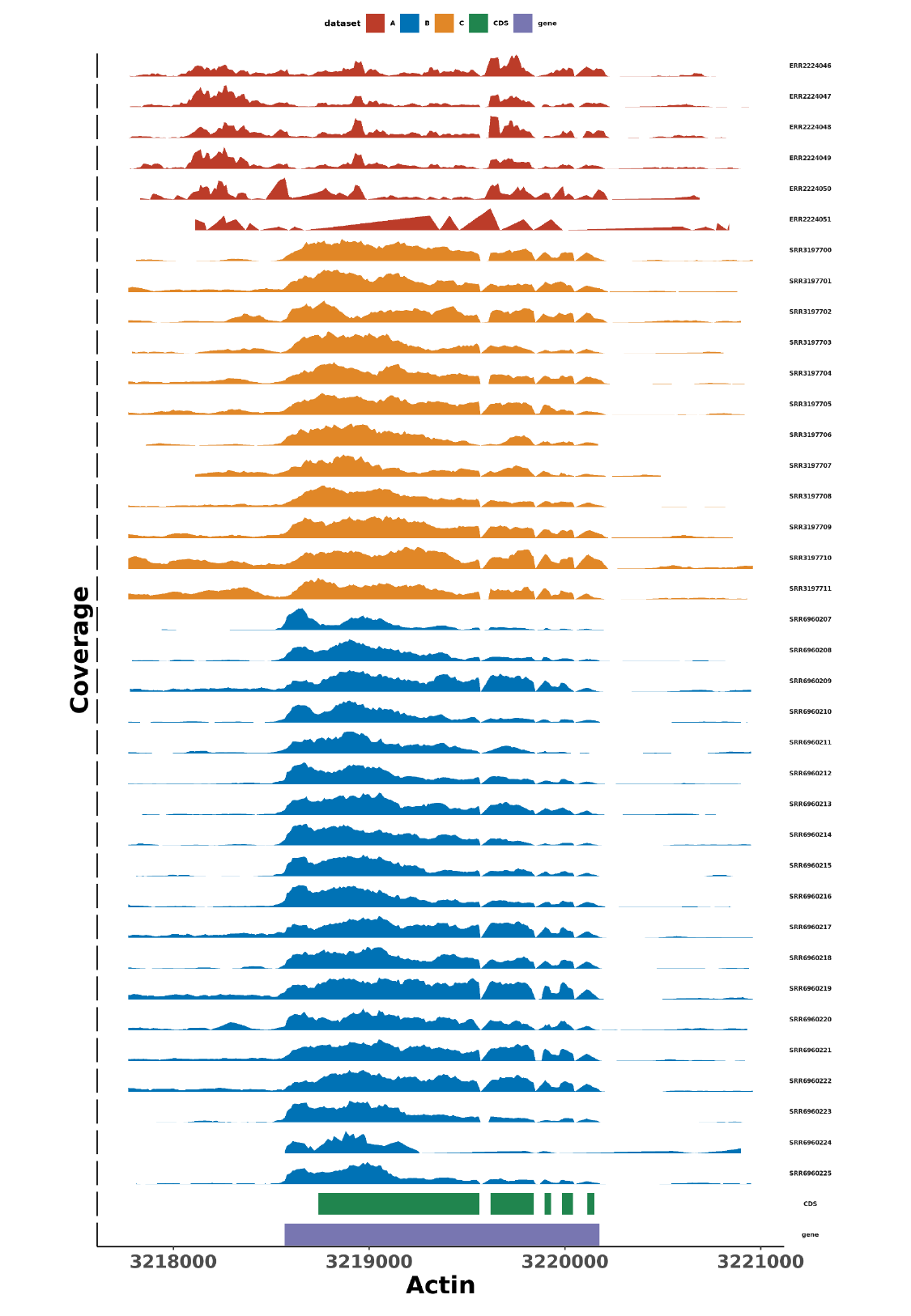

## Slide 3
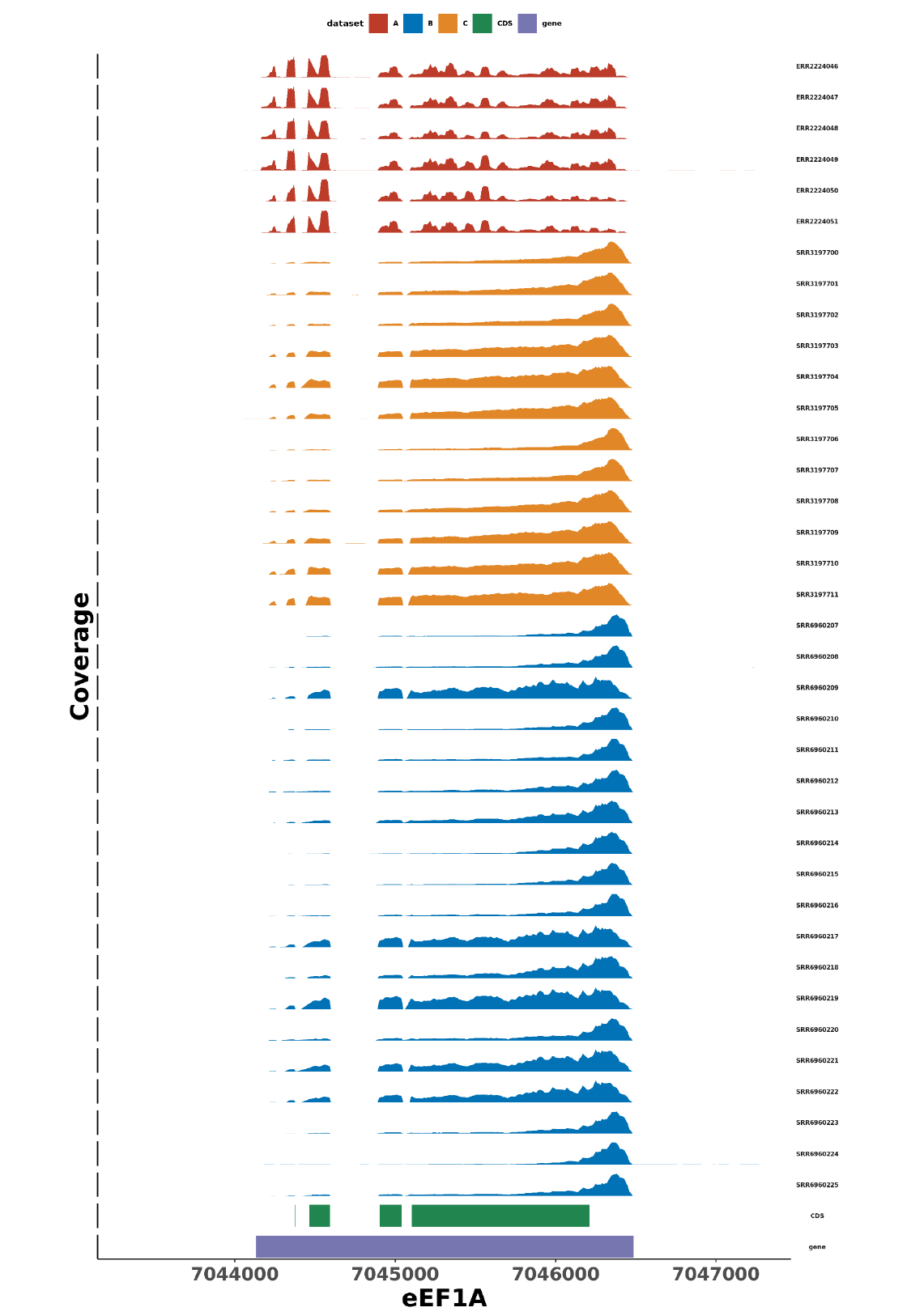

## Slide 4
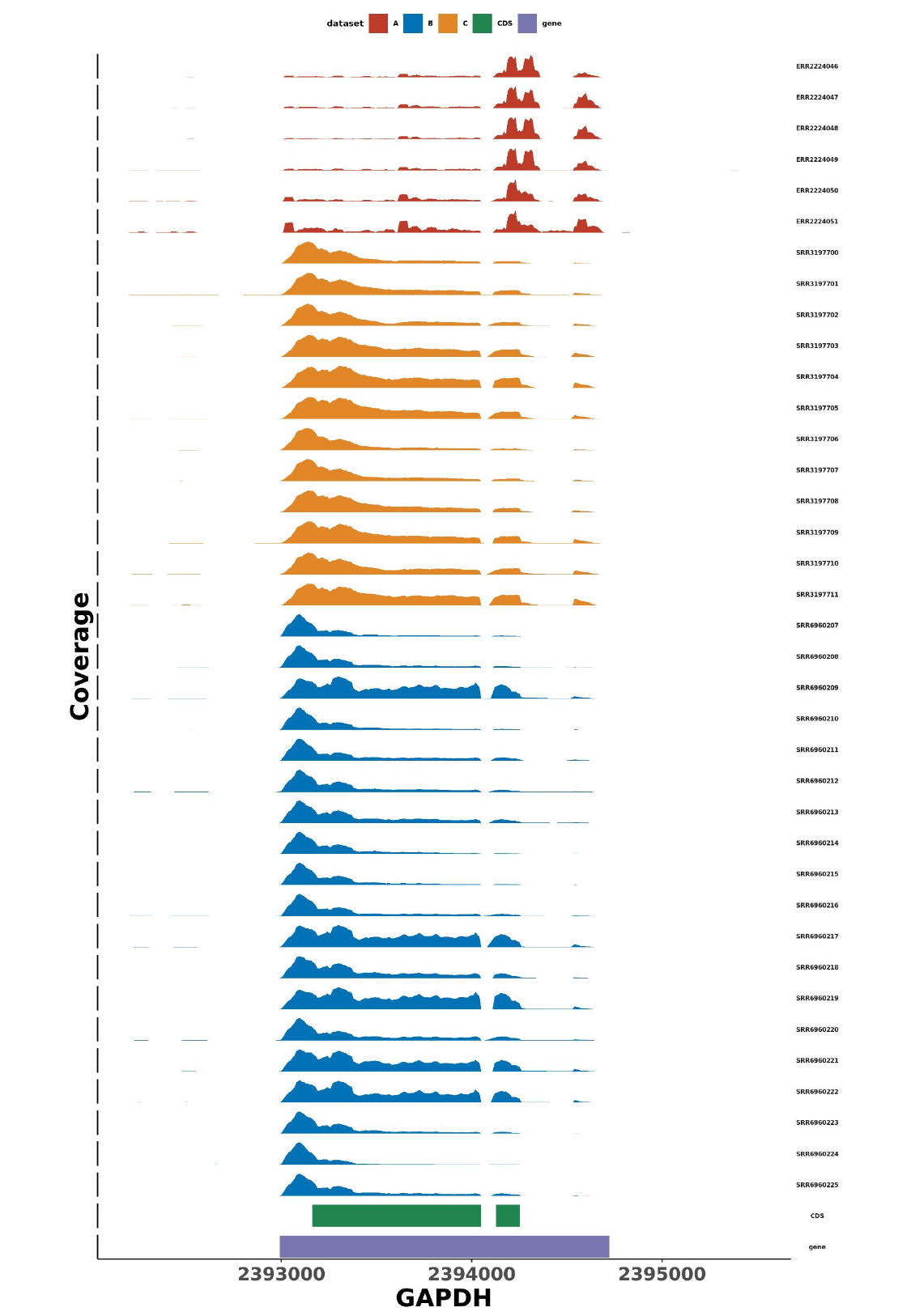

## Slide 5
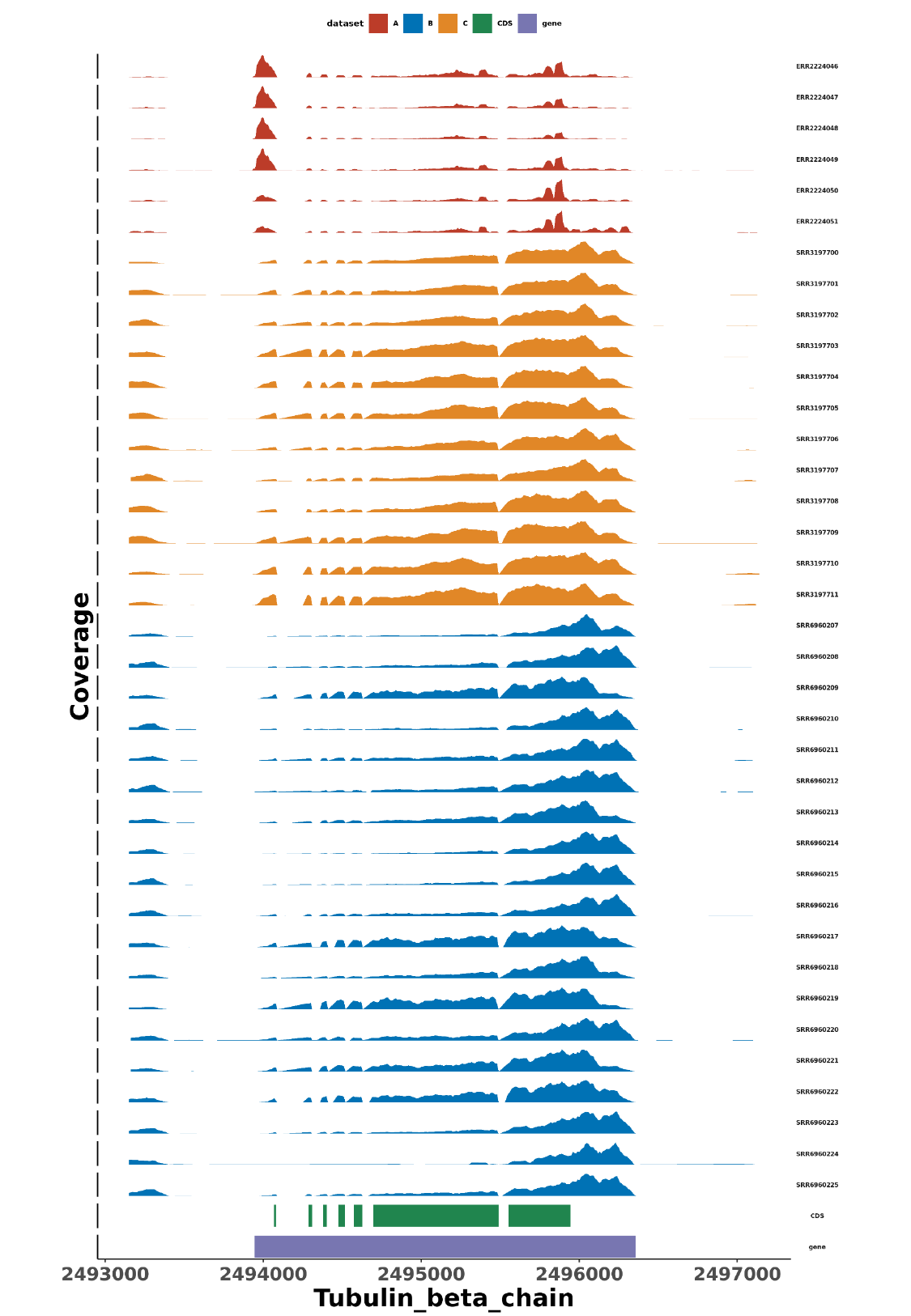

Supplement: Supplementary file 5 — Additional file 5. (PPTX 737 kb) [file 12864_2022_9085_MOESM5_ESM.pptx]
